# Generation of an iPSC line (CRICKi001-A) from an individual with a germline *SMARCA4* missense mutation and autism spectrum disorder

**DOI:** 10.1016/j.scr.2021.102304

**Published:** 2021-05

**Authors:** Liani G. Devito, Lyn Healy, Shehla Mohammed, Francois Guillemot, Cristina Dias

**Affiliations:** a Human Embryo and Stem Cell Unit, The Francis Crick Institute, London, UK; b Department of Clinical Genetics, Guy's and St Thomas' Hospital, London, UK; c Neural Stem Cell Biology Lab, The Francis Crick Institute, London, UK; d Department of Medical and Molecular Genetics, School of Basic & Medical Biosciences, King's College London, UK; e Department of Clinical Genetics, Great Ormond Street Hospital for Children NHS Foundation Trust, London, UK

## Abstract

Germline missense mutations in the BAF swi/snf chromatin remodeling subunit SMARCA4 are associated with neurodevelopmental disorders, including Coffin Siris Syndrome (CSS). Here, we generated an induced pluripotent stem cell line from a male patient with atypical CSS features and a *de n*ovo heterozygous missense mutation in the *SMARCA4* gene (c.3607C>T, p.(Arg1203Cys)). Hair root derived keratinocytes were reprogrammed using non-integrative Sendai virus vector delivery of pluripotency factors. iPSCs generated display normal morphology and molecular karyotype, express pluripotency markers and are able to differentiate into the three germ layers.

## 1 Table 1. Resource Table

**Table:** 

Unique stem cell line identifier	CRICKi001-A
Alternative name(s) of stem cell line	NA
Institution	The Francis Crick Institute
Contact information of distributor	lyn.healy@crick.ac.uk, cristina.dias@kcl.ac.uk
Type of cell line	iPSC
Origin	Human
Additional origin info	Age: 5-9 years Sex: Male Ethnicity: European
Cell Source	Hair root derived keratinocytes
Clonality	Clonal
Method of reprogramming	Non-integrating SeV-mediated delivery of OCT4, SOX2, c-MYC and KLF4 (Cytotune 2.0 Kit, Thermo fisher Scientific)
Genetic Modification	Yes
Type of Modification	Congenital *de novo* mutation
Associated disease	Coffin-Siris Syndrome 4 (OMIM#614609); Autism Spectrum Disorder
Gene/locus	*SMARCA4*
Method of modification	NA
Name of transgene or resistance	NA
Inducible/constitutive system	NA
Date archived/stock date	*December 2019*
Cell line repository/bank	https://hpscreg.eu/cell-line/CRICKi001-A
Ethical approval	This study was approved by the London – Camden and Kings Cross Research Ethics Committee, Ref. 17/LO/0981

## 2 Resource utility

Mutations in *SMARCA4* are associated with intellectual disability (most commonly Coffin-Siris Syndrome) and Autism Spectrum Disorder (Kosho et al., 2014). We report the first patient-derived iPSC resource available for use as a disease-specific cellular model to help elucidate the molecular mechanisms underpinning *SMARCA4* neurodevelopmental disorders.

## 3 Resource details

Mutations in several subunits of the BAF swi/snf chromatin remodelling complex have been associated with a broad spectrum of neurodevelopmental disorders. Heterozygous missense mutations and in-frame deletions in the SMARCA4 helicase subunit have been reported in individuals with a mild phenotypic spectrum of Coffin-Siris Syndrome, characterised by developmental delay, coarse facial features and hypoplastic distal phalanges (Kosho et al., 2014). Germline loss of function *SMARCA4* mutations are a rare cause of autosomal dominant Rhabdoid tumor predisposition syndrome.

Here we report the generation and characterization of an iPSC line derived from an 8 year old patient with autism spectrum disorder, laryngomalacia and mild craniofacial features of Coffin-Siris Syndrome, without digital anomalies. The patient harbours a heterozygous *de novo* missense variant in the c-terminal helicase domain of *SMARCA4*; c.3607C>T; p.(Arg1203Cys) identified on whole exome sequencing (The Deciphering Developmental Disorders, 2015). Having presented with global delay of early developmental milestones, the patient achieved important developmental gains in childhood; at age 9 his general cognitive abilities were within the low normal range for age. He had autism spectrum disorder, dyslexia and dyspraxia.

**Fig. 1:**
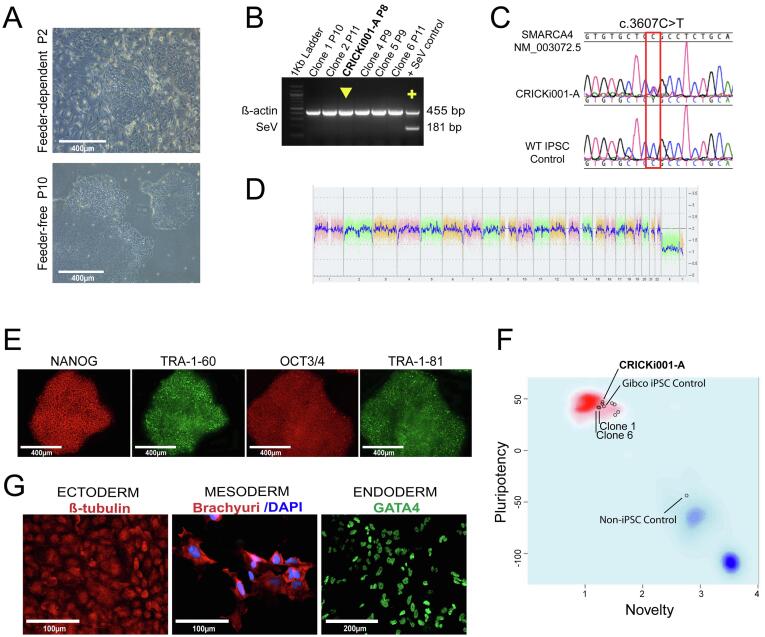
Characterization of iPSC line CRICKi001-A.

Patient keratinocytes were generated from hair root (Cocks et al., 2014) and reprogrammed into iPSCs using non-integrating Sendai virus vectors (CytoTune-iPS, Thermo Fisher Scientific Inc.) expressing human pluripotency factors KLF4, OCT4, SOX2, and C-MYC (Re et al., 2018). Six days after reprogramming, cells were passaged onto inactivated CF1 mouse embryonic fibroblasts (MEFs). Colonies with a typical pluripotent stem cell morphology were individually and manually selected to establish clonal feeder-free iPSC lines (Fig. 1A).

Cells showed typical iPSC morphology after several passages (Fig. 1A). Silencing of expression of exogenous Sendai viral vector was confirmed for clone CRICKi001-A by RT-PCR from passage 8 (Fig. 1B). Dideoxynucleotide sequencing confirmed the *SMARCA4* c.3607C>T mutation (Fig. 1C). Copy number variation analysis by chromosomal microarray indicated a male individual with no chromosomal aberrations (Fig. 1D, Table 2). Stem cell identity of the CRICKi001-A clone was confirmed by expression of pluripotency markers OCT4, NANOG, TRA-1-60, and TRA-1-81 on immunohistochemistry (Fig. 1E) and by gene expression assayed by GeneChip array for PluriTest (Thermo Fisher Scientific) analysis of pluripotency and novelty scores at passage 13 (Fig. 1F, Table 2).

**Table 2:** Characterization and validation.

Classification	Test	Result	Data
Morphology	Microscopic photography	Normal morphology at passage 2 (feeder-dependent) and passage 10 (feeder-free)	Fig. 1 panel A
Phenotype	Qualitative analysis Immunocytochemistry	Staining of pluripotency markers: OCT4, NANOG, TRA-1-60, and TRA-1-81	Fig. 1 panel E
	Quantitative analysis Pluritest	Pluripotency and novelty scores confirm pluripotent state for CRICKi001-A and two independent clones	Fig. 1 panel F
Genotype	CNV and SNP analysis: Karyostat assay (Thermo Scientific) with resolution >2 Mb for chromosomal gains and >1 Mb for chromosomal losses	Male individual; no chromosome aberrations compared to reference dataset (passage 13)	Figure 1 panel D
Identity	STR analysis	16 loci and Amelogenin tested, 100% match between parental keratinocyte and cell line DNA (passage 13)	Submitted in archive with journal

Mutation analysis (IF APPLICABLE)	Dideoxynucleotide Sequencing	Heterozygous for *SMARCA4*, c. c.3607C>T	Fig. 1 panel C
Microbiology and virology	Mycoplasma	Mycoplasma testing by RT-PCR Negative	Not shown; available with the author; submitted in archive with journal
Differentiation potential	*In vitro* differentiation	Directed differentiation to three germ layers confirmed by immunostaining for lineage-specific markers (passage 13).	Fig. 1 panel G
Donor screening (OPTIONAL)	HIV 1 + 2 Hepatitis B, Hepatitis C	Not Tested	NA
Genotype additional info (OPTIONAL)	Blood group genotyping	Not Tested	NA
	HLA tissue typing	Not Tested	NA

*In vitro* differentiation confirmed the ability to differentiate to all 3 germ layers (Fig. 1 G). Identical genetic identity to the donor of the iPSC was confirmed by short tandem repeat (STR) profiling (Table 2).

## 4 Materials and methods

### 4.1 Hair follicle keratinocyte generation

The donor was recruited and collection of human hair was carried out after informed written parental consent in accordance to the “BUILD Study” Research Ethics Committee (17/LO/0981) approved protocol. Hair root was harvested and cultured as previously reported with modifications (Cocks et al., 2014). Briefly, anagen phase hair roots were plated on Matrigel coated culture dishes, overlaid with a coverslip and cultured at 37 °C with 5% CO_2_ in Advanced DMEM:F12 (Gibco) supplemented with fetal bovine serum and Glutamax I (Gibco). At day 7 media was changed to Complete EpiLife (Gibco) supplemented with HKGS (Gibco) and 10 μM Y-27623 (Tocris). Keratinocytes were cultured for 1 passage before being cryopreserved.

### 4.2 iPSC cell generation and expansion

Thawed keratinocytes at passage 2 were seeded 1.84 × 10^4^/cm^2^ in 2 wells of a 4-well dish coated with Coating Matrix (Gibco). Cells were reprogrammed 2 days post-seeding, using the CytoTune-iPS 2.0 Sendai Virus Reprogramming Kit (ThermoFisher) according to the manufacturer’s instructions, with modifications (Re et al., 2018). Cells cultured in EpiLife supplemented with HKGS and 5 mM Y-27362 were transduced with the viral vectors at MOIs reported in (Re et al., 2018) and placed in a humidified incubator at 37°C, 5% CO_2_. The next day the media was replaced with media without viral vectors. Six days post-infection cells were passaged onto previously inactivated MEFs and transferred to a hypoxic incubator at 37 °C, 5% CO2, 5% O2. The medium was progressively switched over 4 days from day 13 to 100% KSR medium (Advanced DMEM(Gibco)/Knock-Out Serum Replacer (Gibco)/Glutamax (Gibco)/2-Mercaptoethanol (Gibco)/4ng/ml FGF 2 (Gibco)) with 10µM Y-27362.

After the emergence of iPSC-like colonies, those with appropriate morphology were manually picked on day 28 and transferred to Matrigel coated 6-well plates with mTeSR1 medium (StemCell Technologies) containing 10µM Y-27362. Medium was changed after 24 hours. Colonies were expanded by splitting at 1:3 to 1:6 ratio every 4-6 days and maintained in a hypoxic incubator at 37 °C, 5% CO2, 5% O2.

### 4.3 Pluritest analyses

Using the gene expression array PluriTest assay (ThermoFisher), genome-wide transcriptional profiles of the hiPSC line clones were compared to an extensive reference set of previously characterized induced and embryonic pluripotent stem cell lines.

### 4.4 Immunostaining

Pluripotency potential and differentiation were evaluated by immunostaining, performed as previously (Devito et al., 2018), with modifications. Undifferentiated and differentiated cells were washed twice with DPBS (Ca2+, Mg2+, Thermo Fisher Scientific) prior to fixation with 3.7% paraformaldehyde (Sigma-Aldrich) for 20 min at room temperature (RT). Cells were permeabilized with 0.5% Triton X-100 (Sigma-Aldrich) for 5 min at RT then incubated with primary antibodies (Table 3) overnight at 4˚C. The following day cells were washed twice with DPBS and incubated with secondary antibody (Table 3) for 30 min.

**Table 3:** Reagents details.

Antibodies used for immunocytochemistry/flow-cytometry			
	Antibody	Dilution	Company Cat # and RRID
Pluripotency Markers	Mouse anti-TRA-1-60	1:100	Millipore Cat# MAB4360, RRID: AB_2119183
	Goat anti-NANOG	1:100	R&D Cat# AF1997, RRID: AB_355097)
	Mouse anti-TRA-1-81	1:100	Millipore Cat# MAB4381, RRID: AB_177638
	Mouse anti-OCT4	1:100	Santa Cruz Biotech Cat# SC-5279, RRID: AB_628051
Differentiation Markers	Goat anti-GATA-4	1:100	R&D System Cat# AF2606, RRID: AB_2232177
	Mouse anti-βIII-tubulin	1:100	Sigma Cat# T5076, RRID: AB_532291
	Goat anti-Brachyury	1:100	R&D Cat# AF2085, RRID: AB_2200235
Secondary antibodies	Donkey anti-mouse Rhodamine IgG	1:100	Jackson Immunoresearch Cat# 715-295-150, RRID: AB_2340831
	Donkey anti-goat FITC IgG	1:100	Jackson Immunoresearch Cat# 705-095-147, RRID: AB_2340401
	Donkey anti-rabbit FITC IgG	1:100	Jackson Immunoresearch Cat# 711-095-152, RRID: AB_2315776
	Donkey anti-mouse Alexa Fluor 488 IgM	1:100	Jackson Immunoresearch Cat# 715-545-140, RRID: AB_2340845

Primers			
	Target	Forward/Reverse primer (5′-3′)	
Sendai Virus (qPCR)	SeV	5’-GGATCACTAGGTGATATCGAGC/-3’-ACCAGACAAGAGTTTAAGAGATATGTATC	
Targeted mutation analysis	*SMARCA4*	5'-CAGAGGCCACCTTCCCTTTT/3'-CTCACCTCATCCTGCTCCTC	

### 4.5 iPSC differentiation into three germ layers

iPSC were direct differentiated into the three germ layers, -endo, -meso and -ectoderm, using the STEMCELL Trilineage Differentiation kit (STEMCELL Technologies) per manufacturer’s instructions. Expression of the lineage-specific markers (Table 3) was assessed by immunostaining as described above for lineage-specific markers at day 5 (mesoderm and endoderm) and day 7 (ectoderm).

### 4.6 Dideoxynucleotide sequencing

PCR amplification (primers listed in Table 3) using the Q5 Hot Start High-Fidelity polymerase (New England Biolabs) was performed on cell line genomic DNA extracted using the QIAamp DNA Micro Kit (Qiagen).

### 4.7 Chromosomal microarray

Array comparative genomic hybridization (CGH) using the KaryoStat assay (ThermoScientific) was performed on iPSC genomic DNA.

### 4.8 Reverse transcription PCR analysis of SeV vectors

RNA was extracted at multiple passages using the RNeasy mini Kit (Qiagen) and cDNA prepared using the SuperScript IV First-strand cDNA synthesis kit (Invitrogen). Presence of remaining SeV vectors was tested by RT-PCR using SeV-specific primers (Table 3).

### 4.9 Short tandem repeat (STR) profiling

STR profiling on the DNA from the parental sample and iPSC line was performed by the Francis Crick Institute Cell Services STP using the Powerplex 16 HS system (Promega).

### 4.10 Mycoplasma detection test

The absence of mycoplasma contamination was confirmed by the Francis Crick Institute Cell Services STP using the Universal Mycoplasma Detection Kit (ATCC 30-1012K) for PCR amplification.

## Declaration of Competing Interest

The authors declare that they have no known competing financial interests or personal relationships that could have appeared to influence the work reported in this paper.
